# Outcomes of Laparoscopic vs. Open Reversal of Hartmann’s Procedure: A Single Centre Experience

**DOI:** 10.7759/cureus.17242

**Published:** 2021-08-17

**Authors:** Zehong Chen, Nandu Nair, Umar Hanif

**Affiliations:** 1 Trauma & Orthopaedics, Sandwell General Hospital, Birmingham, GBR; 2 General Surgery, Royal Stoke University Hospital, Stoke-on-Trent, GBR; 3 Trauma and Orthopaedics, Northampton General Hospital, Northampton, GBR

**Keywords:** hartmann procedure, reversal of hartmann's procedure, laparoscopic reversal hartmann's procedure, colostomy, minimally invasive laparoscopy

## Abstract

Introduction

Hartmann’s procedure is widely performed to fix colonic obstruction and perforation. It should ideally be followed by a reversal to restore bowel continuity. Reversal of Hartmann’s procedure was traditionally performed using an open technique. However, in recent days, the use of a laparoscopic approach has become increasingly popular. In our retrospective observational study, we aim to investigate the outcomes of laparoscopic versus open reversal of Hartmann’s procedure in a UK tertiary centre.

Methods

All patients who underwent reversal of their Hartmann’s procedure between January 2017 and December 2019 were included in the study. Data including demographics, days between primary operation and reversal, laparoscopic or open reversal, length of hospital stay following reversal procedure, 30-day readmission, mortality, and complication rate were collected. Statistical analysis was performed using t-test and chi-squared test.

Results

Forty-nine patients underwent reversal of Hartmann’s procedure from January 2017 to December 2019. The mean age of our cohort was 59.6 ± 13.2 years. There was no significant difference in baseline demographics of both groups, apart from the number of days between the primary operation and reversal procedure. There was also no statistical difference in length of stay, 30-day readmission, and mortality between laparoscopic and open reversal techniques. However, there was a higher incidence of wound complications in patients who underwent open reversal of Hartmann’s procedure.

Conclusion

The reversal of Hartmann’s procedure is a challenging operation. We found no significant difference between both open and laparoscopic approaches, but our study might be confounded by various factors including small sample size and selection bias. A larger, randomised study with greater statistical power is needed to confirm our findings.

## Introduction

Hartmann’s procedure was first described by Henri Hartmann in 1921, as a novel approach in response to high rates of complications and mortality following resection and primary anastomosis of left-sided colonic obstruction [1]. One century later, this procedure is still widely performed to fix colonic obstruction and perforation in patients, both in elective and emergency settings. Hartmann’s procedure involves resection of the rectosigmoid colon, closure of the rectal stump, and creation of an end colostomy. Over the years, indications for the procedure have grown to cover a wide range of abdominal pathologies including colonic malignancy, diverticulitis, ischemia, and volvulus [2].

Hartmann’s procedure should ideally be followed by a reversal to restore bowel continuity. Reversal of Hartmann’s procedure involves the closure of a left-sided colostomy and anastomosis of a proximal colon and distal rectal stump. Patients with successful reversal of Hartmann’s procedure often report better quality of life, as they no longer need to manage the physical and psychological challenges associated with a stoma [3]. Despite the benefits of reversal of Hartmann’s procedure, many patients do not proceed with it and instead, remain with a permanent colostomy. This is due to a variety of reasons including high rates of post-operative complications such as chronic infection, adhesion, anastomotic leak, technical challenges during the reversal such as difficulty in identification of and anastomosis to a short rectal stump, patient’s comorbid status limiting further surgery and patient’s preference [4,5]. With these considerations, patients are often carefully selected for the reversal procedure and less than half of all patients who undergo a Hartmann’s procedure will end up having a reversal [6]. The United Kingdom (UK) National Bowel Cancer Audit in 2019 reported that 89.7% of patients who underwent Hartmann’s procedure still had a stoma at 18 months postoperatively [7].

Reversal of Hartmann’s procedure was conventionally performed using an open technique. In recent days, laparoscopy has become increasingly popular due to its benefits in postoperative recovery and surgical outcomes [8,9]. In our retrospective observational study, we aimed to investigate the outcomes of laparoscopic versus open reversal of Hartmann’s procedure in a UK tertiary centre.

## Materials and methods

Data collection

All patients who underwent reversal of their Hartmann’s procedure in our hospital between January 2017 and December 2019 were included in the study. Patients who did not have all of their parameters recorded in the hospital electronic database were excluded from the study. 

Variables

The following parameters were recorded for all patients: age, gender, American Society of Anaesthesiologist (ASA) grade, co-morbidities, number of days between primary operation and reversal of Hartmann’s procedure, laparoscopic or open reversal, length of hospital stay following reversal procedure, 30-day readmission, mortality, and complication rate. Two independent authors corroborated data collection.

Outcomes

Our primary outcomes were 30-day readmission and 30-day mortality rates. The secondary outcome was the length of hospital stay.

Statistical analysis

All analyses were intention-to-treat, hence patients whose laparoscopic reversal of Hartmann’s were converted to open were analysed in the laparoscopic arm. Statistical analysis was performed using GraphPad Prism version 9.1.2 (GraphPad Software, San Diego, CA, USA). Continuous data are presented as mean ± standard deviation and analysed using a t-test. Categorical data are presented as numbers and percentages and analysed using a chi-squared test. Data was considered statistically significant when p < 0.05. 

## Results

Forty-nine patients underwent reversal of Hartmann’s procedure from January 2017 to December 2019. Patients were routinely followed-up but duration of follow-up was not analysed as part of the study. The mean age of our cohort was 59.6 ± 13.2 years. 53% of patients were males. The most common indication for Hartmann’s surgery was complicated diverticulitis (51%) followed by colorectal cancer (27%). Twenty-seven patients (55%) underwent an open reversal while 22 patients (45%) had a laparoscopic reversal. Of the latter, nine patients (41%) required conversion to an open reversal. 

There was no statistical difference in baseline demographics of both groups, apart from the number of days between the primary operation and reversal procedure. The average duration between the initial Hartmann’s surgery and laparoscopic reversal was 682 ± 446.3 days, compared to 447 ± 269.5 days in the open reversal group (p = 0.04). Baseline characteristics are presented in Table 1.

**Table 1:** Baseline characteristics of patients undergoing reversal of Hartmann’s procedure

Characteristic	Laparoscopic reversal n=22 (%)	Open reversal n=27 (%)	P-value
Age (years)	58.8 ± 13.4	60.1 ± 13.3	0.73
Gender, male	13 (59)	13 (48)	0.57
Indication for Hartmann’s procedure			
Colon cancer	8 (36)	5 (19)	
Diverticulitis complication	10 (45)	15 (56)	
Perforation	1 (5)	2 (7)	
Trauma	0	2 (7)	
Others	3 (14)	3 (11)	
Open Hartmann’s procedure	15 (68)	22 (81)	
Mean ASA score	2.1	2.1	0.73
ASA score			
1	2 (9)	3 (11)	
2	15 (68)	16 (59)	
3	5 (23)	8 (30)	
Co-morbidity			
Diabetes	6 (27)	2 (7)	0.12
Ischaemic heart disease	2 (9)	6 (22)	0.27
Hypertension	6 (27)	9 (33)	0.55
Days between operation	682.4 ± 446.3	444.7 ± 269.5	0.04
Conversion to open	9 (41)	-	

There was no statistical difference in short-term outcomes of laparoscopic or open reversal (Table 2). Five patients from each group were readmitted to hospital within 30 days. Complications requiring readmission in the laparoscopic reversal group included high output ileostomy, intra-abdominal collection and retained sutures. Complications in the open reversal group requiring readmission included anastomotic leak, renal failure due to high output ileostomy and three incidences of wound complications. There was no mortality within 30 days. 

**Table 2:** Short-term outcomes in patients undergoing reversal of Hartmann’s procedure

Characteristic	Laparoscopic reversal n=22 (%)	Open reversal n=27 (%)	P-value
30 day re-admission	5 (23)	5 (19)	0.74
30 day mortality	0	0	-
Length of hospital stay (days)	10.2 ± 7.6	11.7 ± 9.9	0.45

## Discussion

Duration between primary Hartmann’s procedure and reversal

There is no clear consensus with regard to the optimal time for reversal of Hartmann’s procedure. In the existing literature, the average duration for reversal is seven to eight months after the primary Hartmann’s procedure [10-13]. Contrastingly, our study reported an average duration of 22 months for laparoscopic reversal and 14 months for an open procedure. 

Delayed reversal has been shown to optimise clinical and nutritional states of patients prior to their reversal procedure, and enable resolution of the underlying pathology, besides providing time for adhesion and scar tissues to mature [14,15]. However, prolonged duration to reversal is also associated with increased complications such as atrophy of the distal stump and difficulty locating it during surgery [16,17]. 

It is important to note that an element of selection bias might be introduced in these studies, as surgeons who anticipated challenging reversals might delay the operation for various reasons, including optimising the patient pre-operatively. Additionally, early reversal might not be an option in patients who underwent Hartmann’s procedure for malignancy due to the administration of adjuvant chemoradiotherapy. These patients tend to have a longer interval before consideration of reversal, and a reversal is often performed via an open approach. 27% of our patients had their index Hartmann’s procedure due to colon cancer, which might contribute to our protracted interval to reversal. 

Conversion from laparoscopic to open reversal

Laparoscopic reversal of Hartmann’s procedure is one of the most technically challenging operations in colorectal surgery, and high conversion rates to open surgery have been reported [18]. Our study found a conversion rate of 41%, which is significantly higher than the reported average of 16%, but within the range of 0-50% [19]. 

Various reasons could contribute to a laparoscopic reversal being converted to open; the most common being multiple adhesions, small pelvic workspace, and inability to identify, or injury to the rectal stump. Chouillard et al. also found that the conversion rate to an open reversal was lower if the primary Hartmann’s procedure was performed laparoscopically [20]. This could expound our high conversion rate, as seven out of nine patients (78%) who underwent conversion had an index open Hartmann’s procedure. 

Complications

Laparoscopic reversal of Hartmann’s procedure is a technically difficult operation but has been reported to carry benefits such as decreased complication and mortality rates [8,9]. In our cohort of patients, there was no short-term mortality. There were five cases of 30-day readmission in each group, with an increase in wound complications (11%) in patients who underwent an open reversal. This was again consistent with published literature, which reported higher events of wound complications in patients who underwent open reversal of Hartmann’s procedure [21,22]. 

Outcome measures

Similar to existing literature, there was no statistical difference in our 30-day readmission and 30-day mortality rates between both groups. Our 30-day mortality rate was also comparable to that of existing literature [23-26]. Contrary to Siddiqui, Yang, and Morgan, laparoscopic reversal did not reduce the length of hospital stay [26,27].

Limitations

There are several limitations to our study, namely its retrospective nature and our small sample size. The small cohort and low volume of Hartmann’s reversal performed in our centre limited our statistical power and might explain why we were not able to demonstrate statistical effects in our cohort. Selection bias could also be introduced, as the patients and surgeons jointly decided on the surgical approach. Other factors that possibly confounded our study included patient variables such as nutritional status and the variation in laparoscopic technical skills among surgeons, who were at different points of their learning curves. 

## Conclusions

Reversal of Hartmann’s procedure is a challenging operation, and its success is dependent on patient factors and surgeon expertise. There was no significant difference in outcomes between laparoscopic and open reversal of Hartmann’s procedure, although there was a higher incidence of wound complications in patients who underwent open reversal of Hartmann’s procedure. When considering the appropriate approach for reversal of Hartmann’s procedure, surgeons should take into account patient factors such as preference and co-morbidities, and surgeon factors such as prior training and laparoscopic skills. As our study might be confounded by various factors including small sample size and selection bias, a larger, randomised study with greater statistical power is needed to confirm our findings.
